# Supplementary material for: Chromothripsis during telomere crisis is independent of NHEJ, and consistent with a replicative origin
Source: Genome Res. 2019 May;29(5):737–49. doi: 10.1101/gr.240705.118 (PMC6499312; doi:10.1101/gr.240705.118)
Supplement: Supplemental Material [file supp_gr.240705.118_Supplemental_file_1.zip › contigs/annotated_contigs/DB51/contig.2.DB51_length_573_mean_cov_4.5410122164.docx]

**DB51_length_573_mean_cov_4.5410122164**

```
CAATTAGACAGAAGTCTAGGCAGTGTTAGTTTGGAGGGGAAAGACACCAGATGGGAGGCCGTTGAGCTGAGATTTTTGGGTAACCTTAA
 >chr6:158469955-158470153 + E=2e-107
GTCTGGTAGGAGTGGTGGGGTGCAGGGGAGAGGTGTGGAGGTGCTGTCTGCGATCCTTGGGCCTCTTGAGTGCTGATCGAGACTCTGCT

GTGACTTCCACAGC|CAGGAG|CACCCAGATTCATAAAGCAAGTTCTTAGAAAGCTTTAAAGAGACTTAGACTCCTACACAATGACAGC
               >chr6:81126468-81126755 - E=3e-147
GGGAGATTTTAACACCCCACTCACCATACTGGACAGATAATTGAGACAGAATATTTAGGGCCACAGCATAGTACAGCATCAAATAGACC

TGATAGGCATTTATACAACTCTCCACCAAACAAACAAAAAAAGCAAATAAAAACCCAGAATACCTAAAACTTAAAGTATAATAATAATA

AAATTAAAAAAAATAAAAATAAAATAAAAAACCCA|CATTGGAAAACTGTAAAATGATCACATTATCCAAAATAAAAAAATTCCCCGCC

AAAGCAAAAAAATTGAAACCCTAAAAAAAAATTTTTAGGAAA
```
